# How do new doctors prescribe insulin? Qualitative exploration of the complexity of everyday practice and implications for medical education

**DOI:** 10.1136/bmjopen-2025-099128

**Published:** 2025-09-18

**Authors:** Tim Dornan, Ciara Lee, Jason Hancock, Karen Mattick, Hannah Gillespie, Florence Findlay-White, Richard Conn

**Affiliations:** 1 Queen’s University Belfast, Belfast, UK; 2 University of Otago Medical School, Dunedin, New Zealand; 3 University of Exeter Medical School, Exeter, UK; 4 Medical Education, University of Exeter Medical School, Exeter, UK; 5 Newcastle University, Newcastle upon Tyne, UK; 6 Centre for Medical Education, Queen’s University Belfast, Belfast, UK; 7 School of Medicine, Ulster University - Derry Londonderry Campus, Londonderry, UK

**Keywords:** MEDICAL EDUCATION & TRAINING, DIABETES & ENDOCRINOLOGY, Inpatients

## Abstract

**Abstract:**

**Objectives:**

(1) Analyse in depth an exemplar safety-critical task required of newly qualified doctors (prescribing insulin) and (2) Provide transferable insights into how undergraduate education could better educate medical students to meet the demands of practice when they become postgraduate trainees.

**Design:**

Document analysis of doctors’ reported experiences of insulin prescribing, an everyday task that has an emergent logic of practice and harms not just patients but (psychologically) new doctors. Application of third-generation (social emergence) complexity theory to explore why practice can be ‘mutually unsafe’.

**Settings:**

A system of care comprising all five Northern Irish (UK) Health and Social Care Trusts, which together provide healthcare to a population of nearly two million people.

**Participants:**

68 postgraduate year 1 and year 2 trainees (PGY1/2s), mainly PGY1s.

**Main outcome measures:**

Thick description of new doctors’ contexts of action, reasons for acting and specific actions. We present this as a narrative compiling all 68 stories, 13 detailed exemplar stories and a diagram summarising how multiple factors interacted to make practice complex.

**Results:**

Situations that required PGY1/2s to act had interacting layers of complexity: (1) disease trajectories; (2) social dynamics between stakeholders and (3) contextual influences on stakeholders’ interactions. Out-of-hours working and unsuitable wards intensified troublesome contextual influences. All three individually complex layers ‘crystallised’ briefly to create ‘moments of action’. At best, PGY1/2s responded proactively, ‘stretched time’ and checked the results of their actions. At worst, PGY1/2s ‘played safe’ in unsafe ways (eg, took no action), acted on unsafe advice or defaulted to actions protecting them from criticism. Informal, pervasive rules emerged from, and perpetuated, unsafe practice.

**Conclusions:**

New doctors’ work includes acting on indeterminate, emergent situations whose complexity defies rules that are determinate enough to be taught off the job. If new doctors are to perform capably in moments of action, medical students need ample, supervised, situated experience of what it is like to take responsibility in such moments.

**STRENGTHS AND LIMITATIONS OF THIS STUDY**

- Rather than surveying diverse tasks that new doctors are expected to do, as other researchers have tended to do, we chose a single task that new doctors do very often, which can make them very anxious and causes considerable harm to patients.
- Following Lewin’s recommendation that ‘there is nothing so practical as a good theory’, we used social emergence theory to shed practical light on empirical findings.
- We conducted a qualitative document analysis to provide rich, informative detail about new doctors’ everyday practice and used a picture, a summary narrative and 13 individual stories to communicate our findings.
- Rather than moving directly from reading the texts to coding and thematising them, we stayed close to postgraduate year 1 and year 2 trainees’ intact stories and identified chains of cause and effect within them; only then did we identify commonly recurring patterns of complex causality and synthesise a final interpretation.
- As is typical of qualitative research, those strengths bring with them limitations. We have not provided proof of a link between cause and effect for subsequent researchers to replicate or refute. Rather, we offer transferable insights that we hope will help clinician-educators as well as researchers combat the problem of ‘mutual unsafety’.

## Introduction

‘*Rather than making calculated choices between alternatives, (new doctors) often make decisions based on fulfilling their identities and following rules they themselves may not be aware of’.*^1^

Contemporary healthcare education is grappling with a ‘wicked problem’^2^: many doctors’ capability, at graduation, is unequal to the work they face. This can result in physical and psychological harm to patients and psychological harm to new doctors. Despite researchers having approached it from several angles, this ‘wicked’ problem resists even a clear problem statement. Research clarifying the problem and offering new solutions could help achieve ‘mutual safety’^3^ : a more capable, healthy and resilient workforce causing less harm to patients.

The phrase ‘education and training’^4^ acknowledges that two different pedagogies contribute to preparing medical students to practise as doctors. A simple way of distinguishing between these is that education helps future doctors respond creatively to ‘messy’ situations, perhaps by improvising, while training helps them apply ‘best practice’ norms to standard situations. In recent years, authoritative statements^5^,^7^ have pushed educational policy towards requiring students to demonstrate that they have achieved codified performance standards. This emphasis on training may have unintentionally reduced the perceived value of clinical rotations, where education to manage indeterminate situations has traditionally taken place. A potential ‘knock-on effect’ is new doctors being caught off guard when complex interacting circumstances create harmfully indeterminate situations. This offers a tentative explanation for the wicked problem of unsafe practice: perhaps new doctors lack capability because the alluring simplicity of rules dictating how they **should** act is in tension with the indeterminate, negotiated ways that they **must** sometimes act.^8^ Since harm is resisting regulators’, curriculum leaders’ and educators’ best efforts, it is time to question the assumptions that guide contemporary educational policy and practice. To catalyse this, researchers’ need to examine critically what new doctors are expected to do.

One current way of understanding this is ‘clinical decision-making’, which is taught using a priori, rules-based probabilistic logic.^9 10^ Better application of this therapeutic logic reduced the proportion of ‘suboptimal to optimal’ antibiotic prescriptions.^11^ Research into another task commonly performed by new doctors, prescribing insulin for patients in hospital, was less amenable to a priori logic.^3^ So many rapidly changing, interacting factors influence the effect of insulin that the appropriateness of an action sometimes emerges only after the event. Logic, moreover, is not the only factor at play. Harm can emerge when multiple, perhaps illogical factors coalesce and create indeterminate situations where ‘heat-of-the-moment’ emotional reactions trump logic.^9 12 13^ The quotation at the head of this article from Engeström, who has examined doctors’ situated reasoning in fine detail, further endorses the complex social nature of practice.

The concept of clinical uncertainty has provided an informative perspective on clinical decision-making. It too has confirmed that clinicians sometimes ‘feel their way’ through problems.^10^ A complex interplay between clinicians’ attributes (worldview, prior experience etc), features of the contexts where they practise (including systems of care and relationships with others) and clinical emotional, cognitive and behavioural responses can, under certain circumstances, make practice indeterminate. Clinicians may experience a ‘dynamic subjective perception of not knowing what to think, feel or do’.^14^ Perhaps this explains why cognitively informed interventions that use simulation and/or assessment to reduce uncertainty can, under some circumstances, cause trainee clinicians’ to have negative emotional responses.^15^

There has been much research into new doctors’ preparedness for practice, usually concluding that transitions into a practitioner’s identity are turbulent.^16^ As a result, it is recommended that students need to be better prepared, particularly emotionally and socially^17 18^, which means that new doctors need capabilities beyond clinical logic. Research examining experiences of acclimatisation to practice settings has confirmed the complex, social nature of new doctors’ transitions into practice. Transitions are turbulent because the inherent difficulties of clinical tasks, the complexities of sociocultural milieus and the individual capabilities and personalities of new doctors interact.^19^

Available knowledge about new doctors’ preparedness for practice is further complexified by differences in the medical education continuum. There are international differences between the types of individuals entering practice, tasks they are expected to perform, and sociocultural milieus. Important, interacting variables include students’ age and maturity at entry (undergraduate vs graduate entry programmes), the length of programmes (from as little as 3 years to 6 or more years) and whether graduates enter practice within a single-specialty residency (as in the USA and Netherlands) or a generalist residency (as in the UK, Australia and New Zealand). In the extreme case, young (and relatively immature) graduates may work on specialist (eg, surgical) teams, which routinely depute generalist (ie, non-operative) tasks to new doctors.

Perhaps the most influential variable, and the one that is least influenced by relatively fixed variables like age of entry and length of programme, is the balance between off-the-job and on-the-job learning. In the UK, for example, ‘assistantships’ now ensure that students spend a minimum of 10% (and perhaps up to 20%^20^) of their medical programme within practice settings. The norm is higher in the USA and Netherlands and perhaps longest in programmes with immersive longitudinal integrated clerkships. It is not just the amount of time students spend in practice that varies, however, but also the amount of responsibility they experience,^21^ which may be higher for simple tasks than for the most safety-critical tasks.^20^ That variability limits the usefulness of general surveys of preparedness and calls on researchers to explore entry to practice in greater depth.

Problems are wicked when relationships between cause and effect are complex: in ‘wicked’ situations, ‘quick fix’ solutions do not work because multiple causal factors interact with one another and with the open contexts in which they operate. The relevance of complexity to practice is illustrated by research that analysed in depth the experiences and actions of clinicians involved in a single patient’s fragmented care on a transplant unit. Care delivered by clinical teams, the research suggests, can be an emergent property of complexity.^22^ Overall, research into the complexity of practice has been marred by: a general failure to define complexity^23^; reliance on secondary rather than primary research^10 14 15 23^; actions being examined apart from their social contexts; and a dominant uniprofessional conceptualisation of clinical practice, even when it is, in reality, interprofessional.

The purpose of this research was to suggest how educators, students and regulators might better approach education for safety-critical practice. Research into uncertainty, clinical decision-making and preparedness, reviewed above, provided a mandate for us to assume that practice has at least some features of complexity. We set out to represent the complexity of new doctors’ practice from a theoretical stance that leads towards educational solutions. Inspired by the call from the philosopher Husserl^24^ to strip away assumptions and ‘return to the thing itself’, we posed a deliberately open research question: ‘how do new doctors act and why do they act as they do?’. The preceding text and the Methods section that follows explain why prescribing is an educationally relevant action, and why researchers need to choose a clearly defined, informative instance of prescribing. We reasoned that prescribing insulin for hospitalised patients was an informative instance and that applying social complexity theory to rich, empirical data would help us provide transferable insights to the medical education community.

## Methods

### Conceptual orientation to complexity: social emergence theory

Cristancho *et al*’s critique of medical education research^23^ recommended that authors writing about complexity should articulate their underlying assumptions, use clear language and reference appropriate primary sources. Our conceptualisation of complexity was drawn from Sawyer’s social emergence theory.^25^ Complexity thinking, he argues, has been dominated by metaphors drawn from physical and biological systems. His central tenet is that human social systems are different. The ability to communicate using speech, language and other symbols creates greater complexity. Sawyer departs, also, from the logic and metaphors of psychology, which he describes as too individualistic and atomistic to explore human social complexity. In preference, he uses sociocultural theory, which does not dichotomise the individual from the social level of analysis and allows for human interaction to be radically open to external and internal influences.

Those features made social emergence a logical choice for research into how postgraduate year 1 and 2 doctors (PGY1/2s) respond to indeterminate situations. Its assumptions allowed us to examine doctors’ actions as part of a string of processes that emerge from interactions between patients, nurses and doctors in specific contexts. It is well suited to interprofessional practice because it does not attribute activity to individuals or even classes of people. Rather, activity consists of chains of interactions within whole social groups. In addition, the concept of emergence provides a relevant angle on the relationship between education and practice. Billett has explained how, throughout human history, capability to work has emerged from practice and vice versa.^26^ Supervision and instruction play adjunctive rather than primary roles in education for practice.

Applying the preceding logic to prescribing education, medicines and other technologies of clinical care are not sole agents. They act within a nexus of unbounded and sometimes barely visible processes of human interaction and communication. While, ultimately, individuals prescribe insulin, their actions are tempered by socially sanctioned interpretations and meanings. In sum, social emergence theory provides a rationale for examining, in fine detail how and why:

- New doctors act.
- Higher-level properties of processes of care emerge from doctors’ actions.
- Emergent properties of social contexts influence doctors’ actions.

It provides a rationale to scrutinise practice, interpret empirical data from a social constructivist stance and offer insights whose relevance may extend beyond the research context.

### Settings: instances that can provide transferable insights

#### Geographic

Northern Ireland (NI) is one of the four regions of the UK. We conducted the research in all five integrated Health and Social Care Trusts: the Belfast, South-Eastern, Southern, Western and Northern Trusts (part of the UK National Health Service). Postgraduate education across all five Trusts is managed by a regional Deanery. The UK General Medical Council regulates medical education and practice. With few exceptions, only doctors can legally write prescriptions for patients under acute hospital care.

#### Setting within clinical practice: insulin prescribing

We chose prescribing (using pen and ink on paper charts) because PGY1/2s write 70% of prescriptions for patients in UK hospitals; it is one of their most common tasks, and one for which they feel ill-prepared^17^; in-depth research into students’^27^ and PGY1/2s’ experiences^11^ has shown its complexity, particularly during on-call periods when services rely most heavily on PGY1/2s to prescribe.^28^ Even more specifically, we chose insulin prescribing for hospitalised patients because:

- This exemplifies ‘predictive’ clinical logic, whose rightness or wrongness does not exist a priori but evolves over time. The same logic applies to at least one other everyday duty of new doctors (intravenous fluid therapy), which makes it more likely that our findings will transfer.
- One in six patients in UK hospitals has diabetes^29^; many (including some controlled without insulin at home) require insulin in hospital.
- Insulin has a narrow ‘therapeutic window’: too little or too much can have severe, even fatal consequences.
- Because insulin is so often prescribed, senior clinicians usually leave FY1s to do this, often with little supervision.^30^
- Because PGY1s prescribe insulin from the moment they leave medical school and are receiving postgraduate education while they are prescribing it, insulin therapy is an indicator of educational effectiveness.
- A national audit in England and Wales provides reliable audit data about the harm it causes within the UK healthcare system:
  - Each week, over 1% of patients with diabetes need to be rescued from hypoglycaemia and over 3% need treatment for life-threatening hyperglycaemia that occurred after hospital admission.^31^
  - Being diabetic increases patients’ length of hospital stay by 25% and increases the likelihood of being readmitted within 28 days of discharge by nearly 50%.^29^

### Study design

The research procedures and findings reported here stem from a large, regional project whose rationale can best be described by Lewin’s maxim that, ‘if you want truly to understand something, try to change it’^32^ Recently, the wisdom of his thinking has been reflected by the upsurge of complex intervention and implementation science research methodologies. We sought to understand the above-mentioned burden of harm in the expectation that this exemplar would yield insights that could be transferred to other problematic tasks (perhaps most immediately intravenous fluid therapy, as noted above). Online supplemental Information 1 provides details of the ‘Act Wisely’ (AW) programme in which this research was situated. The AW pedagogic toolkit^33^ and a qualitative evaluation of its impact^3^ have been published and an exhaustive set of operating procedures is freely available for readers to inspect.^34^ We highlight here those aspects of the programme that directly affect the validity of this research report:

- The intervention took the form of ‘case-based discussions’ (CBDs), which PGY1/2s requested. These facilitated discussions explored a recent experience of prescribing insulin, which was personally meaningful to the PGY1/2.
- We set up a programme to educate a cadre of doctors, nurses and ‘Patient Advocates’ (see Patient and Public involvement, below) to facilitate confidential, psychologically safe, non-judgemental, reflective conversations.
- All PGY1/2s are required to complete several one-to-one CBDs, when they describe a clinical event in detail and are helped to learn from it.
- By email, word of mouth and messages sent by the Deanery, we offered every PGY1/2 in the Region a CBD, explaining that this might be conducted by a trained nurse, pharmacist, doctor or Patient Advocate, depending on who was available at a time and place that suited the PGY1/2, but all of whom had been trained to follow the same procedures.
- To confirm the appointment for the CBD they had requested, a PGY1/2 had to write an anonymous description of the event they wished to discuss into a proforma. While the contents of the form remained unchanged, its presentation evolved to move from longhand to electronic data recording. Online supplemental Information 2 shows the final, electronic version. For the CBD to count as one of their required training events, the PGY1/2’s notes had to contain detailed clinical and contextual details of the situation.
- Using open questioning and attentive listening, the facilitator helped the PGY1/2 talk through the experience and probed important features of it, annotating the form with extra detail.
- So that the PGY1/2 could concentrate on relating their experience, the facilitator rather than the PGY1/2 wrote clarifying notes about:
  - Who had called the PGY1/2 and why.
  - The patient’s situation.
  - How other clinicians and stakeholders (eg, family members) had been involved.
  - How the PGY1/2 had responded to the situation.
  - How the situation had subsequently unfolded, including how the PGY1/2 had checked their action.
- The facilitator helped the PGY1/2, in light of the discussion, to make one or more Specific, Measurable, Realistic and Time-bound commitments to improve their insulin prescribing behaviour, and to put into words what they had learnt from the experience.
- At the end of the discussion, the facilitator added a reflective note about the trainee’s learning, and about the educational environment.
- One copy of the proforma was sent to the PGY1/2 so they could add to or correct the information (which they rarely, if ever, did) and a second copy was forwarded to the Deanery as evidence of completion.
- A distress protocol was in place to deal with trainees who experienced difficult emotions, though this was never invoked. Facilitators also had the opportunity to discuss their experiences with project leaders if they became distressed, though peer interaction was the main source of support.
- Our online resource centre^34^ and online supplemental information 1 provide further details of the AW programme.

The CBD protocol resulted in facilitators writing detailed and informative reports of trainees’ lived experiences of practice, which provided the materials for a documentary analysis.

### Patient and public involvement

As explained above and in online supplemental information 1, we involved diabetic patients on insulin to be ‘Patient Advocates’: to train as facilitators and conduct CBDs.

### Analytic procedures, including reflexive engagement with the data

We reasoned that a traditional thematic approach, which would parse out every PGY1/2’s intact narrative into coded extracts and then organise codes into cross-cutting themes, was incompatible with our orientation towards complexity. The direct opposite—presenting only whole stories—would be compatible with our complexity orientation, but it would be harder to transfer the findings to clinical situations other than insulin treatment. It seemed most appropriate to explore cause-and-effect relationships within PGY1/2s’ whole stories and then synthesise a representation of clinical complexity that could, in future research and teaching, be further explored and used to influence education and care. To do this, we chose a reflexive approach as proposed by Crabtree and Miller.^35^

All authors were experienced in qualitative data analysis. TD (diabetologist and researcher into workplace learning), CL (had worked as PGY3 in NI; postdoctoral researcher into clinical uncertainty) and FF-W (former diabetes specialist nurse; regional diabetes care adviser) had led and conducted the fieldwork. They contributed ‘insider’ expertise to the analysis. HG (former PGY1 and 2 in NI, trainee nephrologist and postdoctoral education researcher now in England) and RC (NI paediatrician and postdoctoral researcher into education for safe prescribing) had close knowledge of the research context but had not been directly involved. Two team members who had neither been involved in the fieldwork nor worked in NI joined them to contribute an outsider perspective (as regards the research context) to this analysis: JH (psychiatrist whose doctoral studies were in tolerance of uncertainty) and KM (non-clinician scientist; leader of health education research into prescribing, uncertainty and workplace well-being).

TD led and conducted the procedural steps of the analysis. First, he asked all authors to read the content of CBDs provided by 247 PGY1/2s, which we had used to write an earlier report.^3^ Authors did so in three pairs, each reviewing a convenience sample of one-third of this huge, collated dataset. Each pair included someone who had conducted the fieldwork and someone who had not. To open authors’ minds to possible interpretations of the data, TD encouraged them to be aware of their ‘gut responses’ to the stories, not just think analytically. He encouraged JH, KM, RC and HG to use their non-participant perspectives to help pairs be aware of threats to the validity of their reflexive interpretations including: self-serving bias; self-deception; implicit bias; inflexible frames of reference; anchoring in pre-existing knowledge; and substitution (tendency to substitute simpler interpretations when complexity made interpretation difficult).^35^ He asked them to consider the significance of emotions. Participants’ expressed emotions and their own experienced emotions could signal important content, but their own emotional responses were also a potential validity threat. He asked authors first to read their allocated dataset individually, then discuss the data with their pair-mate, using points of apparent disagreement to trigger deeper reading and reflection, asking each pair to synthesise their thoughts into notes to be presented to the other two pairs during an online discussion. Having established common ground between ‘outsiders and insiders’ in this first stage, he reorganised the pairs, so that each author worked with a different pair-mate, two of the three pairs being led by someone who had not conducted the study (to avoid too strong an insider influence). Since quantity could easily overwhelm the quality of analysis, he purposively divided the first 100 of the 247 transcripts between the three pairs and asked authors to extract complete verbatim data and paste them into columns in an Excel spreadsheet reporting what was going on, paying particular attention to:

- How a PGY1/2 became involved in a situation they had chosen to discuss.
- PGY1/2s’ detailed evaluation of situations, including:
  - The patient’s personal as well as clinical needs and wishes.
  - The needs and wishes of other people, particularly whoever had called the PGY1/2 (usually a nurse), but also other health professionals and patients’ family members.
  - Other features of the action context, including demands that were currently competing for their attention, and features (emotions as well as thoughts) that might inhibit them from or predispose them to taking particular actions.
- The action(s) a PGY1/2 took, with particular attention to:
  - How confident they were to act as they did and whether they sought information, advice or other help.
  - Whether they checked their action, either in the moment or later.
  - (If known) the outcome.
  - How they had felt then, and felt now, about their action.

The pairs judged 68 of the 100 cases to contain enough information to contribute to the final analysis. TD then analysed the contents, harmonising the contributions of three pairs with intentionally diverse perspectives into a structure that best represented what all pairs had found, referring back repeatedly to the raw data. He then inserted additional columns in the spreadsheet to identify broad, superordinate dimensions of complexity that the cases represented. He wrote a synthesis of the spreadsheet’s contents and chose individual cases that exemplified how different dimensions related to one another and sketched a diagram representation of findings. CL, KM and RC reviewed his synthesis critically, constantly comparing it with the raw data, identifying misquotations and considering the validity of the dimensions that the interpretation offered. In a final round, all authors reviewed the interpretation and agreed on a final report of results.

### Presentation of results

**Figure 1:**
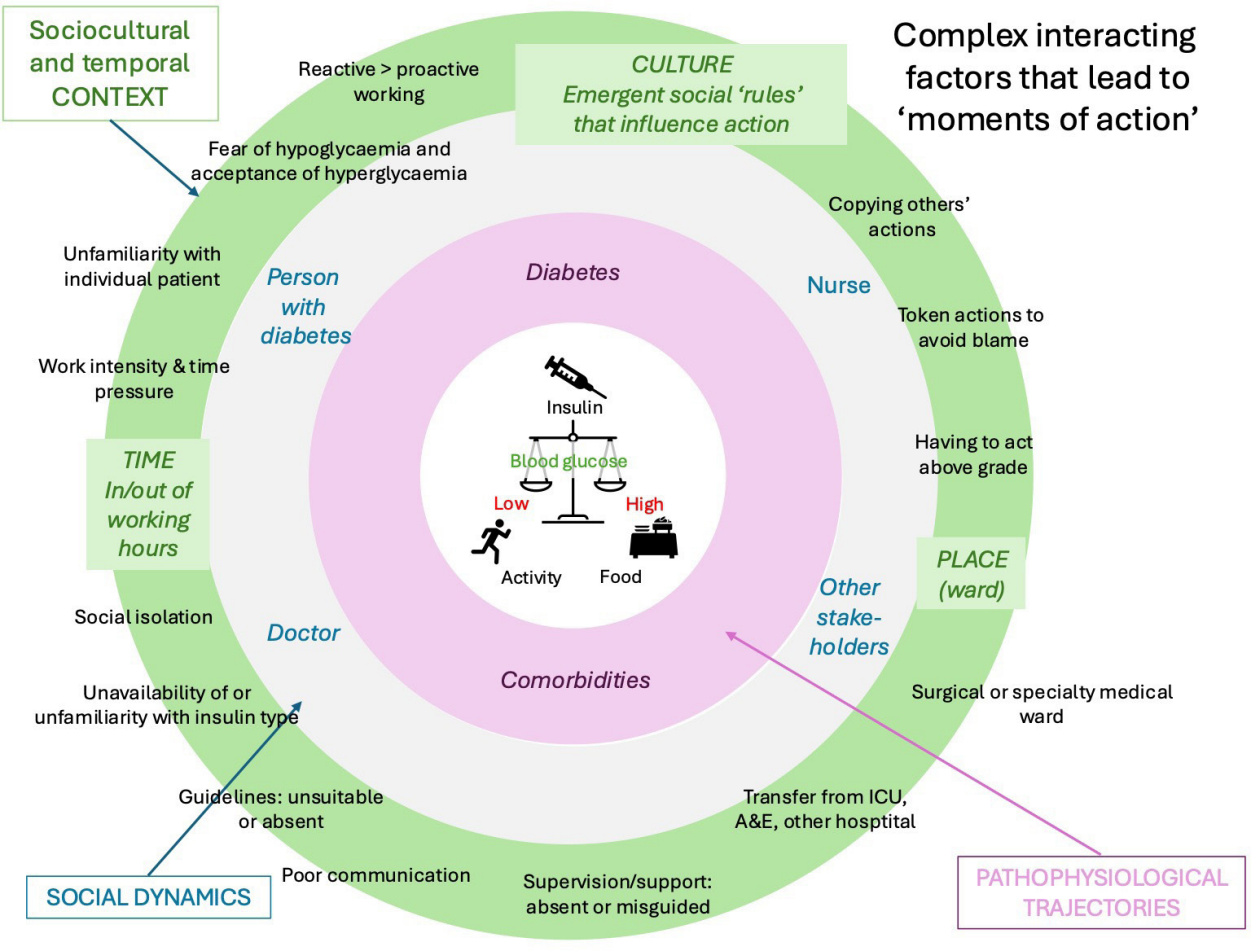
This cartoon illustrates how calls to PGY1/2s ‘froze’ complex, interacting contexts, social dynamics and pathophysiological trajectories to create moments from which action emerged. At the centre is the physiological balance that, in health, uses insulin to regulate a person’s blood glucose level, compensating for activity, food intake and other factors to prevent glucose becoming harmfully low or high. The inner concentric circle depicts factors that become disordered in a person with diabetes who is unwell and admitted to hospital: the trajectories of comorbidities (illnesses that brought the person into hospital and/or developed as a result) and the trajectory of the diabetes itself, influenced by treatment and comorbidities. The middle concentric circle depicts the triad of person with diabetes, doctor and nurse (and sometimes other clinicians), interactions between whom influence treatment. The outer concentric circle depicts three classes of factor (culture, time and place), and specific instances of each class that influence the social dynamics of patient care. Any factor in any circle can interact with any factor in the same, or other circle. PGY1/2s, postgraduate year 1 and year 2 trainees.

We present the results in three ways. To optimise the transferability of our findings to other safety-critical situations, the main results section condenses the findings into a categorised narrative and Fig 1 summarises them pictorially. Finally, as is most consistent with complexity thinking, box 1 presents a set of narratives that exemplify the diversity of situations and response. TD wrote a compiled and condensed version of each of 13 stories that all authors had agreed best exemplified the findings. He asked coauthors to identify misrepresentations. box 1 The results section references individual PGY1/2s’ cases using the abbreviations NPx (Narrative PGY1/2x) when an exemplary PGY1/2 is presented in box 1 and Px when, due to space constraints, their narrative is not included in this report.

We put in place the following safeguards against deductive disclosure of PGY1/2s’ identities:

- Their data were identified only by unique study numbers; their names, genders, ages and specific work locations were not disclosed to researchers analysing the data.
- We removed any site or subspecialty information that could identify individuals from the stories.

## Results

The final dataset included the stories of 62 PGY1s and 6 PGY2s drawn from all 5 Trusts. Their reflective conversations had been facilitated on four occasions by nurses, five occasions by patient advocates, 21 occasions by doctors and 39 occasions by pharmacists (on one occasion, by a nurse and pharmacist together). Box 1 is a text box, presenting 13 exemplar narratives of action. Figure 1 is a diagrammatic synthesis of the findings.

### The beguiling simplicity of calls to action

Typically, a nurse initiated action by calling a PGY1/2 to write or alter one or more prescriptions for insulin, ‘review’ or ‘sort out’ a patient’s diabetes, correct an unduly high or low blood glucose level, or start insulin. Apparently simple calls drew PGY1/2s into complex interactions (NP21). Other calls acknowledged complexity: to mediate between patient and nurse when the patient’s usual type of insulin was unavailable (P21), or assess a non-specifically unwell diabetic patient (NP35). PGY1/2s might be called to take prompt, sometimes immediate action, for situations that had evolved slowly but been overlooked; for example, a delayed insulin dose.

### Factors influencing action

#### Context: dominant effects of time and place

##### Time: the heightened complexity of out-of-hours working

PGY1/2s’ memorable experiences typically occurred overnight and/or at weekends (out-of-hours) when PGY1/2s were in double jeopardy because low staffing levels both made them busier and gave them more responsibility.

##### Time: trajectories

PGY1/2s were called to act, not on static situations, but on time-bound trajectories of change: a patient’s diabetes might fluctuate between hypoglycaemia and hyperglycaemia because of suboptimal management (NP21) or for no obvious reason (P44). Hyperglycaemic trajectories, the commoner type, might be acute (NP47, NP51) or chronic (NP15, NP21). Hypoglycaemia might be profound and/or recurrent, perhaps because a patient’s pathophysiology was trending towards lower insulin requirements (P3, P10). The trajectory of a patient’s treatment might need to be changed; for example, from intravenous to subcutaneous insulin after an episode of ketoacidosis (P48). Hospital admission, per se, altered patients’ diabetes trajectories (P50). Being hospitalised reduced patients’ activity and changed their nutrition. Meals (P36) and/or insulin doses might have been forgotten (P41). An insulin dose might not have been prescribed (NP32) or an insulin regimen might be poorly matched to what a patient could eat (P61, 62). There might be no management plan (P31) or records might not have charted the trajectory sufficiently (P51). Trajectories might be upset by inconsistent actions (P11) or by consistently inappropriate actions, as in case P53 where systematic underdosing to avoid hypoglycaemia caused life-threatening hyperglycaemia.

The trajectories of comorbid illnesses added complexity by interacting unpredictably with diabetes trajectories: a patient might be variably unable to eat or drink (NP22, NP47) due to a varying level of consciousness, gastrointestinal surgery (P12), enteral, or parenteral (P13) nutrition, or a patient might surreptitiously take energy drinks (34). Infective or inflammatory disease (P3, 4, 5), severe illness requiring intensive care (P10, 12, 39), kidney failure (P34, 37, 56, 67) and corticosteroid therapy (many instances) tended to increase patients’ insulin requirements while adrenal insufficiency reduced requirements (P46).

##### Place: non-specialist wards

Placement of patients on wards where appropriate medical and nursing expertise was lacking (notably surgical wards (many cases), but also subspecialty medical ones (P9)) further confounded situations on which PGY1/2s had to act.

### Dynamics of social interactions

PGY1/2s responded to patients’ pathophysiological complexity within complex triadic relationships between doctor, patient and nurse.

#### Nurses’ needs and wishes

There were differences, sometimes contradictions, between how nurses and doctors enacted their supposedly shared objective of caring for patients. Nurses were members of relatively stable ward-based communities of practice, which met all the care needs of defined pools of patients longitudinally following well-established handover and documentation procedures. Nurses called PGY1/2s when changes in patients’ conditions required medical action. Particularly out-of-hours, PGY1/2s had different accountabilities from nurses because they were peripatetic, socially isolated members of ad hoc medical hierarchies (dictated by on-call rotas), responsible for large ill-defined pools of patients with whom PGY1/2s interacted on a ‘need-to-act’ basis. Differences between doctors’ and nurses’ accountabilities made tensions inevitable. NP26, for example, describes a nurse barely tolerating a PGY1/2’s need to assess a patient before writing an overdue prescription. NP21 describes a PGY1/2 wanting, for medical reasons, to lower blood glucose with a larger insulin dose when a nurse wanted a smaller dose because they were afraid the patient might become hypoglycaemic. A nurse might ask a PGY1/2 to prescribe a whole batch of insulin doses for different patients, which compiled a doctor’s accountability for treating several complex patients into a rushed exercise of reading and writing charts.

#### Features of patients

There were also tensions when doctors and nurses, who were professionally accountable for delivering care, disregarded patients’ habitual accountability for self-care or when patients preferred to treat themselves in clinically undesirable ways: wishing to remain hyperglycaemic, despite its risks (P17); not wishing to receive insulin, despite needing it (P64); not reliably self-administering insulin prescribed for them; or insisting on managing themselves in ineffective ways (P50). PGY1/2s could find themselves ‘piggy in the middle’ when nurses called them to resolve such tensions.

### Ingrained habits creating an unsafe culture of practice

Some behaviours were so deeply encultured that they became implicit rules, pervading practice contexts, making inappropriate actions legitimate, and making appropriate actions illegitimate. Avoiding hypoglycaemia at all costs and regarding the hyperglycaemia that resulted as an acceptable consequence was one (NP36): this could result in life-threatening hyperglycaemia (NP51). Reactive management was also encultured (NP11). This made it legitimate for experienced doctors working during the day not to make difficult proactive prescribing decisions, leaving less experienced, busy, on-call colleagues to manage patients reactively. A typical knock-on effect was for patients’ blood glucose levels to fluctuate, sometimes widely (NP21). Another suboptimal behaviour was not to prescribe insulin or prescribe only a small token dose (NP11) if a patient’s blood glucose was not currently high, which was unwise because it was likely to cause a harmful upturn in the patient’s glucose trajectory. Another was not to give larger doses than others had given before. Motivated by fear of causing hypoglycaemia, this created conditions for patients to receive insufficient doses repeatedly and for their glucose to progress from being fluctuant to being persistently high and, potentially, decompensating faster than corrective treatment could remedy (P23). The culture that emerged from these practices tended to regard unsatisfactory blood glucose control as an irremediable feature of diabetes rather than a remediable consequence of clinicians’ actions.

Figure 1 illustrates pathophysiological, social and contextual features that affected PGY1/2s’ actions, as described below.

### PGY1/2s’ actions

Box 1 shows how PGY1/2s responded when all the preceding complexity crystallised in deeply encultured, time-poor, busy milieus to create moments of action. At best, they managed patients proactively and checked the effects of their actions, sometimes ‘stretching time’ to do so. Sometimes, though, PGY1/2s ‘played safe’ in unsafe ways, acted on unsafe advice, defaulted to actions that protected them from criticism, or took no action at all. Prior experience helped, particularly when PGY1/2s had gained this under supportive supervision, and most of all when educative supervision had given them agency to act more independently in future. It is striking that their actions were more improvised than trained and rehearsed, and it is striking also how safe action sometimes resulted from the right person being in the right place at the right time. There are informative absences from the dataset: when facilitators explored reasons for having acted as they did, PGY1/2s rarely volunteered that undergraduate or postgraduate education had helped them to act and they were as likely to criticise written guidelines and official sources of information as to say these were useful resources.

Box 1 **Stories of action.**

**How and why PGY1/2s acted**

We chose these narratives and the wording of the headings that organise them to present the spectrum of actions that PGY1/2s took, progressing from reactive to proactive, but with many permutations. The commentary highlights factors in each narrative that influenced PGY1/2s’ present and perhaps future actions.

**1. Inaction**

***P12**. Because a bed was needed on the intensive care unit in the middle of the night, a diabetic patient recovering from surgery for bowel obstruction (who had several other complicating diseases) was transferred to a general ward. This required the patient to be switched from an intravenous insulin infusion to subcutaneous insulin. No medical handover took place. Not knowing how to initiate insulin in this context, P12 left the prescription till the morning.*

Here, circumstances beyond a PGY1/2’s control—the need to take an action they did not know how to take—resulted in their inaction, which left a sick diabetic patient in the precarious position of having no insulin prescribed.

***P26**. Overnight at a weekend, P26 was called to prescribe an insulin dose to be given the next morning. The patient, whom P26 did not know on a ward they did not know, did not have a stable insulin regimen and, despite repeated earlier corrective doses of insulin, remained moderately hyperglycaemic. Neither the patient nor nursing team members could give any information to guide P26’s action, and P26 was instructed impatiently to ‘just prescribe’. Before P26 was able to do so, they were called urgently to manage a sick patient on another ward and were not able to return, perpetuating the pattern of ‘just-in-time’, reactive management.*

In the case of P26, inaction was the accidental result of competing work pressures. Had they not been called away, the appropriate action—planning and initiating proactive treatment—might have been beyond P26’s capability, so continued reactive management was all but inevitable.

**2. Default action**

***P21**. A middle-aged insulin-treated man came to the Emergency Department (ED) hyperglycaemic. He received his (supposedly) normal pre-dinner insulin dose and was moved to a ward early evening. Later, he became hypoglycaemic. Towards the end of the night shift, he was, again, hyperglycaemic so a nurse called P21. On arrival, other urgent tasks having delayed P21, a nurse told them to prescribe corrective insulin without delay. Doubting the wisdom of this, P21 contacted their supervisor. Meanwhile, information emerged: 1) the man had not been given an evening meal in ED; 2) the insulin dose given there consisted, wrongly, of only fast-acting insulin.*

P21 had to respond to the request of a nurse, whose forceful behaviour may have reflected frustration about P21’s delayed arrival. P21’s action—calling their supervisor for advice— accidentally ‘bought time’ for vital information to emerge. Had P21 acceded to the nurse’s request, it is likely that their action would have perpetuated fluctuation of the patient’s blood glucose between two harmful extremes. The action of not giving extra insulin whilst continuing to monitor the patient closely, a wisely conservative policy, emerged by default rather than intent.

**3. Senior advice perpetuating unsuitable action**

***P11**. A patient with diabetes and an infective illness had erratically fluctuating blood glucose levels. A middle-grade doctor advised P11 against prescribing insulin unless the blood glucose level was moderately high, which set a pattern of different doctors prescribing insulin reactively when called, which did not improve the patient’s glucose fluctuations, and culminated in the patient becoming severely hypoglycaemic several days later. Only then did an endocrinologist advise on the patient’s management, which the middle-grade doctor found educative.*

The middle-grade doctor’s advice implicated P11 in prolonged, suboptimal management that had an adverse outcome. Belated expert advice, though, provided a learning opportunity for both doctors involved.

***P7.** A patient who had been admitted with severe hyperglycaemia remained moderately hyperglycaemic. Unable to discern any pattern that could explain the problem and intimidated by having to take action that could cause harmful hyper- or hypoglycaemia, P7 did not know what to do. They called their middle-grade cover, who prescribed a single insulin dose and advised P7 to ask a nurse to recheck the patient’s blood glucose, but did not manage the situation proactively. The middle-grade doctor did not behave warmly towards P7 and said they had acted ‘by instinct and experience’. P7 learned nothing useful and remained intimidated by diabetes. The patient’s diabetes remained poorly controlled.*

This case illustrates Section 1 of discussion, where it speaks of ‘tacit rules’. The middle-grade doctor’s ‘instinct and experience’ conforms to the ‘rule of reactive management’, which was probably the commonest suboptimal practice, sanctioned by repetition.

**4. Acting safe’ compromising patient safety**

***P51**. A patient who had been hospitalised with DKA was transferred back to a general ward on a rather complex subcutaneous insulin regimen. They became mildly hypoglycaemic during the day but their blood glucose level subsequently rose. Written information about the regimen was ambiguous and it was unclear whether the patient had received their prescribed evening insulin dose. A nurse called P51 mid-evening because the patient’s blood glucose had become moderately high, in response to which P51 prescribed a smaller insulin dose than the one that had earlier caused hypoglycaemia. P51 was called again at midnight because the patient’s blood glucose was continuing to climb. They repeated the dose they had given earlier. Blood glucose remained high but did not rise further. Next morning, the patient was again in mild DKA.*

The small insulin doses P51 prescribed were understandable, given that the patient had earlier been hypoglycaemic, but the tacit rule to avoid hypoglycaemia may have been an influence. In the event, the patient exited from the therapeutic window in an upwards rather than downwards direction. This narrative illustrates how complexity bedevils ‘safe’ clinical practice, because an actor may be ‘damned if they do, and damned if they don’t’.

The narratives analysed in subsections 1–4 have emphasised the constraining effect that complexity had on PGY1/2s’ actions. There is another side to the story. The following subsections describe how, in various ways, PGY1/2s acted resourcefully despite or even because of working in a culture that encouraged short-termism that deferred rather than solved problems.

**5. ‘Stretching time’ to act**

***P25**. After a busy morning’s work, a nurse caught P25 as they were about to go for lunch, asking for insulin to be prescribed for a hyperglycaemic patient. P25 assessed the patient, chose a correction dose of insulin, explained this to the patient, asked the nurse to recheck the patient’s glucose in 30 minutes and deferred changing the patient’s regular prescription pending the result of the later test. P25 referred the patient to a diabetes specialist nurse because it seemed the patient was not injecting themselves correctly.*

By contrast with earlier narratives, where patients might have benefited from PGY1/2s using more of their moment of action to reflect on the call, P25 made time, at personal expense, and used it to engage with a patient and nurse, and craft a proactive management plan, including future checks.

***P47** was called at night to review a patient on an insulin infusion, who was having hypoglycaemic episodes, which may have been explained by the rate of insulin infusion remaining fixed when the patient’s blood glucose level was changing. So P47 prescribed a variable rate insulin infusion. A more senior doctor reversed this decision the following day. Called to the same patient the next night, P47 was asked rather forcefully by members of the nursing team to change the patient back to a variable rate infusion at once. Despite feeling isolated and vulnerable, they did not act at once but took the patient’s records to a room where they could review all relevant information, including practice guidelines in force in the hospital. Only then did P47 agree to the nursing team’s favoured action and switch the patient back to variable rate insulin. P47 felt anxious about this because they were now abandoning a regimen that a more senior doctor had prescribed. He considered calling the middle-grade doctor on call but decided against this because he know how busy they were. P47 did, though, go back twice during the shift to check that the patient was well.*

Several features of this second instance of ‘stretching time’ are noteworthy. Unlike P25, this took place out of hours, when pressures were relentless. It is a longitudinal narrative, where a patient’s management provoked conflict, repeated experiences of which encouraged a PGY1/2 to make time stand still and evaluate the situation thoroughly before acceding to a nurse’s request. As a junior doctor contradicting the action of a more senior member of a hierarchical profession, the complexity within which P47 acted had cognitive, social, and emotional dimensions. This narrative exemplifies how reflective medical practice (36) can address the many dimensions of potentially harmful complexity.

**6. Checking as a component of action**

***P36**. A member of a ward nursing team asked P36, on a Saturday morning, to cancel a dose of fast-acting insulin for a patient who had not eaten breakfast. P36 checked the patient’s glucose level and, finding it high, said that the patient should have insulin. This created conflict and a nurse only agreed to give insulin if P36 reduced the dose. This disagreement sowed doubt in P36’s mind, who became anxious that the patient might become hypoglycaemic (as they had been 48 hours earlier). P36 reviewed the patient 3 hours later and found, to the contrary, that the patient was hyperglycaemic, suggesting that the forced compromise had been to the patient’s disadvantage.*

This narrative shows how constraints on a PGY1/2 elicited negative emotions, despite which they took proactive action. The action was to check the patient’s progress and confirm that the action they had taken under duress was less than ideal and created conditions for more proactive management.

**7. Managing insulin therapy proactively**

***P35**, who had treated a patient’s ketoacidosis a week earlier, was called to see the patient again on a Saturday afternoon because they were ‘restless and unsettled’ having been treated earlier in the day for high blood glucose. P35 already knew the patient, which boosted their confidence. They asked a nurse to measure the patient’s ketones and blood glucose level, which remained moderately high. They prescribed a corrective dose of insulin and increased the patient’s evening insulin dose. They asked the nursing team to monitor the patient’s glucose and ketones hourly.*

This narrative has features in common with P47 (the value of already knowing a patient) and P36 (checking) but illustrates a PGY1/2 going beyond ‘containing’ a situation to managing it proactively.

***P32** was called on a night shift by a nurse who had noticed that a patient’s long-acting insulin had not been prescribed, which had resulted in the patient not receiving it for the last 24-hours. P32 felt confident to manage the situation because they had learned from an endocrinologist on a previous occasion when a similar problem arose. The endocrinologist had opportunistically taught P32 how best to manage this situation, which had stimulated P32 to do some additional online learning.*

This narrative builds on Narrative 35, which shows how prior experience helped PGY1/2s act, and Narratives P11 and P7, by showing how experience of optimal practice, explained ‘on the job’ by an expert, educated PGY1/2s to be more proactive.

***P5**, who had never before managed hyperglycaemia, was called on a Saturday to see an elderly patient with diabetes complicated by urosepsis whose diabetes had been treated reactively with one-off insulin doses but remained hyperglycaemic. Having checked with their middle-grade cover, P5 prescribed a dose of quick-acting insulin and asked the nursing team to measure the patient’s blood glucose hourly and call back if the situation was not improving. P5 also increased the patient’s long-acting insulin and asked their consultant to review the patient’s progress.*

Here, a PGY1/2 was proactive, despite lack of prior experience, illustrating the value of supportive supervision and teamworking, which allowed a PGY1/2 to ask ‘their’ consultant to check the patient’s progress with a more expert eye.

***P31.** After a patient was admitted to hospital because of a hypoglycaemic reaction, a doctor stopped the patient’s insulin treatment without writing any plan for the patient’s subsequent management. This resulted in the patient’s blood glucose being persistently high. After five raised blood glucose measurements, P31 was called to manage the situation. They evaluated the patient’s clinical condition, read the case record, spoke to members of the nursing team, did a further blood glucose measurement, and recorded what they had done. They did not prescribe insulin immediately but asked to be called if the patient’s clinical state deteriorated and went to review the patient next morning. It was now clear that further hypoglycaemia was unlikely, and the patient’s main risk was from hyperglycaemia so P31 restarted the patient’s insulin at a slightly reduced dose, which they subsequently increased.*

This is the most complete example of exemplary, proactive working.

PGY1/2s, postgraduate year 1 and year 2 trainees.

## Discussion

### Principal findings and meaning of the study

We found that ‘moments of action’ emerged from the complexity of practice. Tacit rules emerged from and guided practice, constraining the actions that PGY1/2s could take in such moments. Concisely stated, as complexity thinking demands, our principal finding is that: new doctors needed to examine the interactions between pathophysiological, contextual and social factors from which moments of action had emerged and, taking that complexity into account, act in ways that favoured greatest benefit and least harm. This finding is consistent with recent research, which explored how complex, interacting factors could facilitate or inhibit new doctors’ agency in such moments.^36^ The educational meaning of this research is that curricula and pedagogic processes should address the situated complexity of medical work, starting in the undergraduate phase of the medical education continuum. ‘Scientific’ teaching tends to communicate general truths, or laws. Narrative education, in contrast, communicates evocative instances, at the heart of which lie transferable wisdom. Our findings endorse Calman’s advocacy for narrative education in the form of storytelling and humour.^37^ An important meaning of the findings is that we should educate students in the complex social art of medicine alongside its complex physical basis in science, together providing a rounded education in medical complexity.^38^

### Strengths and limitations

A strength is that this research complements a growing body of knowledge drawn from research into clinical decision-making, uncertainty and preparedness for practice. The most original methodological aspect of our work is the coupling of a situated, qualitative approach with a conceptualisation of complexity that is new to this field. Published two decades ago, Sawyer’s social emergence theory^25^ has been used in healthcare education research little, if at all. This helped us address a dataset that gave novel insights into what new doctors do. Our methodology allowed us to make few a priori assumptions about new doctors’ actions, which increases the likelihood of the findings transferring to other settings and facets of healthcare education and practice.

Our findings have numerous limitations. It would sometimes be more appropriate to approach complexity research from the angles of uncertainty and decision-making, which we did not do because we wanted to shed new light on the more fundamental concept of clinical emergence. It would be appropriate to answer related, but different, research questions quantitatively or to use a different qualitative methodology such as grounded theory, phenomenology or discourse analysis. Our subjective, self-report methodology was, inevitably, a ‘way of not seeing’ as well as ‘a way of seeing’. In particular, we acknowledge that accessing participants’ experiences’ through facilitated reflective discussions, and asking facilitators to record them in writing, may have introduced ‘noise’ into the dataset, although the diversity of facilitators’ backgrounds makes systematic bias unlikely. Although resonance between social emergence theory and our findings strengthens our grounds for proposing that some healthcare education should be orientated towards social emergence, we can only do so cautiously given that our findings came from one task in one phase of the education continuum in one region of one nation.

### Implications

The most far-reaching implication is that contemporary policy, which seeks to quality-assure education by reliably measuring predefined learning outcomes (the UK National Prescribing Safety Assessment in the specific instance of prescribing^39^) is an incomplete solution to the complex practice it seeks to improve. Hippocrates founded the medical profession on the precept that prosocial values would help doctors balance an intrinsic motivation to benefit patients against the possibility of harming them. For millennia, making a commitment to that complex duty motivated doctors to uphold professional standards. Some, though, did not do so and others caused serious harm despite well-intentioned efforts. This focused recent educational policy on patient safety, which made it logical for regulators to take a ‘carrot and stick’ approach: extrinsically motivating doctors not to be ‘unprofessional’. Our findings show that medicine is still a complex practice, to which Hippocrates’ thinking remains relevant, that fear of being deemed unprofessional can cement unsafe habits into practice cultures, and (as suggested previously^24^) that some new doctors are resourceful more despite than because of their education.

By design, this research had little to say about attributes of PGY1/2s. One important factor, though, declared itself: prior experience, particularly under the guidance of a supportive senior practitioner, helped some PGY1/2s act in resourceful ways. Traditionally, medical students learnt to practise during prolonged periods of supervised practice. Billett has shown how novices learn complex practices by participating alongside more experienced practitioners.^26^ Now, though, the pendulum, at least in the jurisdiction where we conducted this research, has swung towards simulated and assessed competence, perhaps augmented by education in entrustable professional activities. A clear implication is that regulators and educators should reconsider policy. There is widespread concern that clinical services are too stretched for inexperienced students to gain supervised responsibility before they qualify. And yet it seems contradictory that, when the main threat to healthcare delivery is shortage of manpower, students who are on the cusp of starting to practise are deemed unfit to contribute to it. Our published inventory of ‘best practice around the world’ shows how resourceful educators have achieved this^21^: we suggest that policy should take a more holistic approach to patient safety, recognise the harm caused by inexperienced graduates, better integrate education and training and make the transition from student to practitioner less abrupt. Even without change in policy, we urge educators, as we have done, to ‘return to the thing itself’: medical practice, in all its complexity. Prolonged, supervised clinical placements, where senior medical students experience what it is like to find themselves precipitated into a moment of action, have an irreplaceable role in medical curricula, particularly in curricula like the one we investigated that launch medical students directly into generalist practice.

### Unanswered questions

Neither education nor training alone is sufficient. Situationally aware doctors combine standardised and reflective working into a seamless whole, as has been shown, for example, in surgical practice.^40^ The research reported here purposefully investigated a supremely messy situation that precipitates new doctors into moments of action day in and day out. Arguably, medical schools relied too long on education alone. While the move towards standardised training and testing has redressed that balance, our findings suggest that the pendulum may have swung too far and that the ideal balance between education and training, and how the two pedagogies can contribute to the preparation of new doctors for practice, remains an important topic of inquiry for educational research and practice.

## Supplementary material

10.1136/bmjopen-2025-099128 online supplemental file 1

10.1136/bmjopen-2025-099128 online supplemental file 2

## Data Availability

No data are available.
