# Supplementary material for: How do new doctors prescribe insulin? Qualitative exploration of the complexity of everyday practice and implications for medical education
Source: BMJ Open. 2025 Sep 18;15(9):e099128. doi: 10.1136/bmjopen-2025-099128 (PMC12458796; doi:10.1136/bmjopen-2025-099128)
Supplement: online supplemental file 1 [file bmjopen-15-9-s001.docx]

# Supplemental Information 1: The Act Wisely [AW] education programme

This short description is a precis of detailed information available in the AW resource centre (34) and in two earlier publications (3, 33) The basic of premises of AW are that one learns wise practice by a] learning ‘mimetically’ (26) from working alongside wise actors and b] reflecting, in psychologically safe settings, on experience. The term reflection, in this context, refers to Donald Schön’s theory of how practitioners learn to practise by gaining experience in ‘the messy lowlands’ of practice and, by looking back on challenges that confronted them and how they responded, learn with the support of colleagues and others (36). AW uses empowerment principles (42) to move beyond reflection and actively change their future behaviour.

- At its centre, is a heuristic derived from theories and empirical evidence regarding prescribing, behaviour change and clinical error, which encourages practitioners to approach clinical situations by doing the following:
  - **The situation**. Listen: Who wants what and why. Involve the patient: what are their needs and wishes; what complicating factors and comorbidities are relevant? Analyse: What situation are you facing?
  - **Me**. Consider: What makes you more or less capable of managing the situation? How would it be wisest to proceed?
  - **Act**. Are you confident to do so? If *yes* proceed. If *no*, seek information or help? If *not sure*, go through the preceding stages again.
  - **Check.** Now: Was it easy; if *yes*, check yourself; if *no*, ask someone else to check. Later: Who should check, when, why, and how?
  - **Reflect**. Now: How did it go? What features of the situation influenced your action? Follow-up: What was the result of your action? For reflection: Which components of this task made you feel good, bad, or unwise?
- Since reflective learning is greatly enhanced by supportive conversations with others, we recruited and trained a cadre of facilitators. They included doctors specialising in diabetes care, pharmacists, general and specialist nurses, and service users who wished to use their experience of treating their own diabetes with insulin to help doctors learn. Empowerment principles informed the design of training workshops.
- We initially offered reflective case-based discussions only to PGY1 and 2 doctors but later conducted them with medical students, dental residents, pharmacists, and nurses.
- We asked PGY1/2s to choose a single experience of prescribing insulin that was salient to them – it could be one which they felt was successful, unsuccessful, resulted in either benefit or harm to a patient, or was in any other way personally meaningful. We provided a proforma which offered the above heuristic as a way of thinking through and writing notes on the experience.
- In the phase of the programme from which these findings were drawn, we conducted CBDs face to face, on-site, wherever in the region the trainee was working. Later, we moved conversations to an online platform.
- During the conversations, facilitators used active listening techniques to help PGY1/2s describe what happened in fine detail, scaffolded by the heuristic.
- At the end of the conversations [not included in this report, but reported elsewhere (3)] facilitators helped PGY1/2s identify learning points and make SMART [Specific, Measurable, Achievable, Realistic, and Time-bound] to one or more behaviours that would enhance their practice.
