# Supplementary figures and images for: How do new doctors prescribe insulin? Qualitative exploration of the complexity of everyday practice and implications for medical education
Source: BMJ Open. 2025 Sep 18;15(9):e099128. doi: 10.1136/bmjopen-2025-099128 (PMC12458796; doi:10.1136/bmjopen-2025-099128)

Supplemental Information 2: Final version of data recording proforma


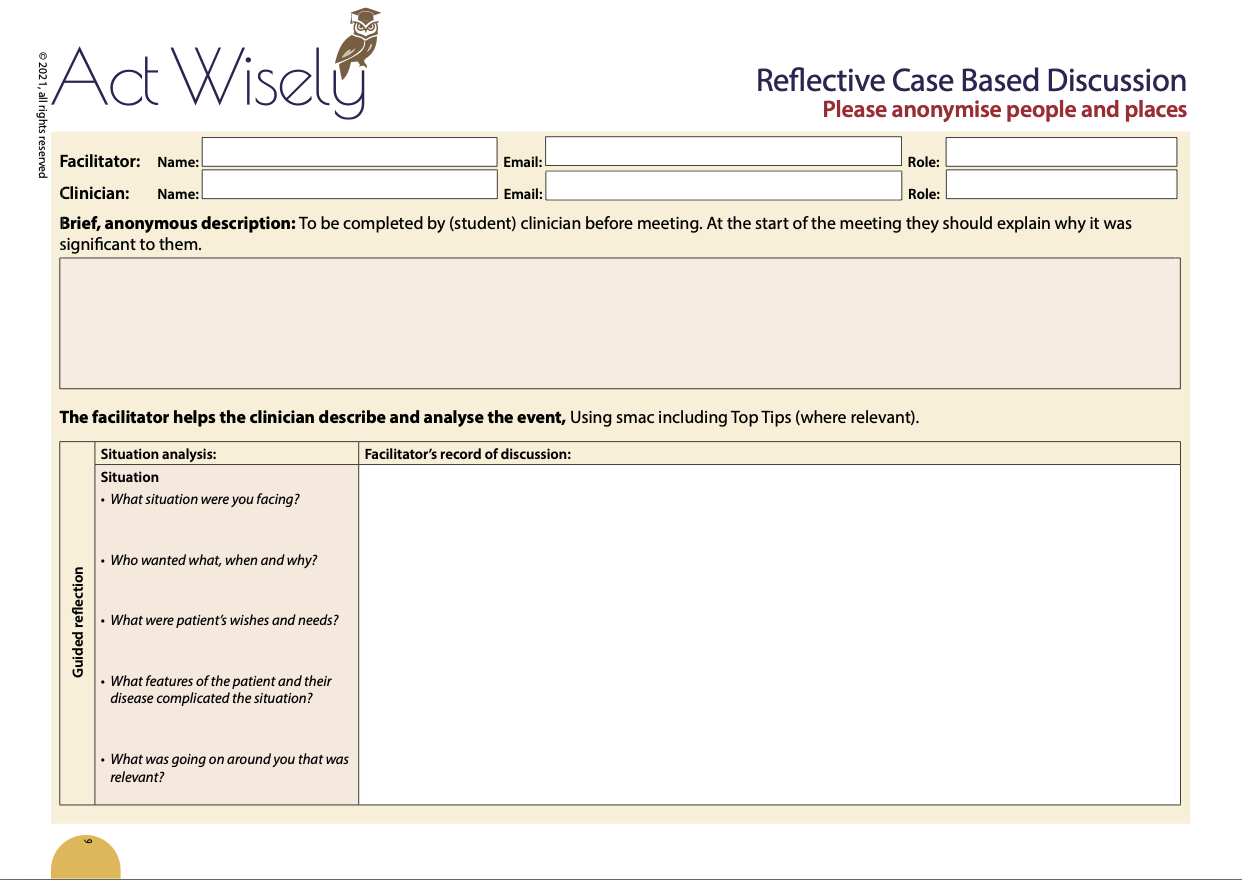


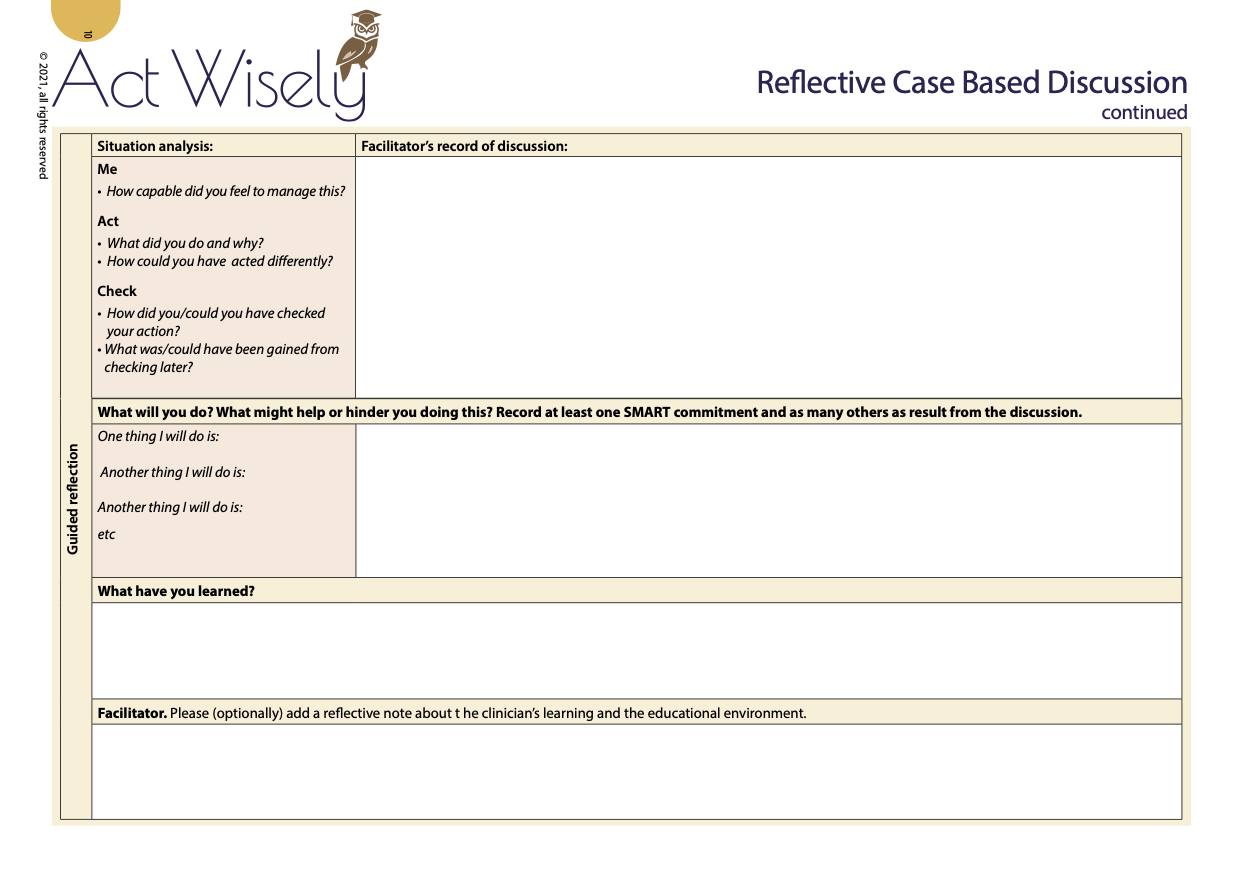

Supplement: online supplemental file 2 [file bmjopen-15-9-s002.docx]
