# Supplementary material for: How do new doctors prescribe insulin? Qualitative exploration of the complexity of everyday practice and implications for medical education
Source: BMJ Open. 2025 Sep 18;15(9):e099128. doi: 10.1136/bmjopen-2025-099128 (PMC12458796; doi:10.1136/bmjopen-2025-099128)
Supplement: Supplementary file 3 [file bmjopen-2025-099128.reviewer_comments.pdf]

PEER REVIEW HISTORY

BMJ Open publishes all reviews undertaken for accepted manuscripts. Reviewers are asked to complete a checklist review form and are provided with free text boxes to elaborate on their assessment. These free text comments are reproduced below.

ARTICLE DETAILS

Title (Provisional)

How do new doctors prescribe insulin? Qualitative exploration of the complexity of everyday practice and implications for medical education

Authors

Dornan, T; Lee, Ciara; Hancock, Jason; Mattick, Karen; Gillespie, Hannah; Findlay-White, Florence; Conn, Richard

---

VERSION 1 - REVIEW

**Reviewer**	**1**
**Name**	**Owen, Katherine**
**Affiliation**	**Warwick Medical School**
**Date**	**16-Apr-2025**
**COI**	**None**

This is an interesting paper exploring how new doctors manage complexity which is pertinent to medical training and preparation for practice. One key area of omission is discussion of the medical assistantship, which is required for all students in the UK by the GMC. The assistantship is designed to achieve just these outcomes, of practical application of knowledge in the messy workplace and preparation for practice. There is a literature surround the assistantship eg Kinston et al 2023 which explores similar themes and should be included. Suggesting that undergraduate medical education needs more constructive alignment with the workplace is a very generalised statement with the variation in curricula- some of which have extended assistantships, some of which use EPAs to engage students with tasks when they have achieved competence. There is a case put forward for patient safety being better addressed through work-place activity over training, simulation and formative assessment, however most educators would consider that moving through these stages is not a replacement for clinical activity, but rather preparation to ensure safe practice.

I think the title should include prescribing/insulin prescribing rather than simply moments of action. With the methodology used the authors comment “we cannot claim that the findings are generalisable” and this should be reflected in the title. Similarly with the objectives in the abstract, the study does not “Provide an in -depth analysis of work that newly qualified doctors do” and this should be more specific.

The study design and methodology are appropriate for the research question.

It is not clear who interviewed the participants- people with lived experience were trained but I’m not clear if it was they who conducted the case based discussions. I would also like to see more clarity on how the discussions were recorded- from the examples given it appears that this was done by the facilitators, however this raises questions for me regarding the implications for an idiographic approach relying on intact narratives. If these narratives are recorded by a 3rd party they are already being seen through a different lens and the strength of the narrative is depleted. If the 3rd parties have different roles and backgrounds this further muddies the authenticity.

I would prefer the details of the precise methods used to be in the body of the text, as this is needed to fully understand the process. The body of the text could be considerably abbreviated.

The relevance of applied mathematics to the theoretical construct is unclear, and the reference given is to a popular science book. For me, this adds nothing and could be excluded. The use of social emergence theory is appropriate as a non-reductionist approach for what are certainly areas of complexity. Clinical decision making is a widely researched concept and this concept could have been applied in more depth. For me the results of this study suggest we should focus more on helping students learnt that clinical decision making is multi-faceted and can include emotional, hierarchical, situational and other factors- some of which impact good decision making and students and newly qualified doctors should be aware of.

The results are clearly expressed and the diagrammatic representation is useful. The examples given demonstrate the different facets impacting on practice and decision making.

References are largely appropriate, but a high proportion (25%) cite the authors own work.

There is certainly merit in this paper, however for me there needs to be more clarity of methods, consideration of the assistantship, reduced and sharpened focus on theoretical models and less reductionist discussion of the current approaches to undergraduate medical education.

Reviewer 2

Name **Hyde, Sarah**

Affiliation **Charles Sturt University, Three Rivers University Department of Rural Health**

Date **01-Jun-2025**

COI **None**

This was a great read, a little challenging in places, and my review provides feedback suggestions it is hoped will improve readability and understanding.

How new doctors learn to respond to complexity

Thank you for the opportunity to review such a novel and theoretically progressive piece of research. I did read this several times and enjoyed the premise of the paper overall, but I also think there are some areas which need more clarity. The paper will provide a new contribution to the literature and is thought-provoking in how we can best teach in ways that are both psychologically safe but still prepare doctors for the complexity of practice in a variety of contexts. The scope was clearly defined, and this is a much- needed area for investigation as workforce demands change, and universities struggle to adapt their teaching to adequately prepare graduates for practicing in contexts with the increased demands and expectations placed on stressed, burnt-out supervisors who are in short supply. My main critique is that the methods still lack clarity and the messaging in the Discussion and Implications were clearer than in the Introduction and so perhaps the authors could think about some re-framing.

Abstract

1. The first objective was sound and I think the paper achieved it. However, the second objective, and all subsequent discussions of curricula alignment in the paper, seem to be quite a stretch to make and I don't think the authors quite achieved the objective "Propose how undergraduate curricula could be more constructively aligned with the demands of medical work". Upon my first reading, this set up an expectation for some clear guidance about curricula, which was not delivered upon. On my second reading, I could see why this objective was stated and the points made in the Discussion about how simulation belies the complexities of practice in 'real-world' setting and may do a disservice in preparing doctors for practice. However, I think the real gem from the discussion was the point made about the education and training continuum and this could perhaps be the basis for reframing the arguments and rather than 'curriculum alignment' per se.

2. The study design is novel and impactful. The use of social emergence (third-wave) complexity theory was interesting and extended my thinking in this space. However, I could not quite follow the use of "analytic logic derived from applied mathematics". I have read the second part of Figure 1 several times, and the paper several times with the purpose to improve my understanding of this, and I still lack clarity and do not see how the applied mathematics logic was used in the narrative analysis. I also could not clearly see what additional value this second paragraph in Figure 1 offered or what this contributed to the research above and beyond social emergence theory. If the authors choose to leave this in, then the links between this method and the analysis need to be made more explicit and the additional value this offered above or in addition to social mergence theory also needs to be clearly articulated. It does involve more work on the reader to try to understand the value of it and that type of work detracts from reading the paper to gain new insights that may improve one's educational practice.
3. The participant numbers are great for a qualitative study of this nature, and I was wondering if they were all UK graduates and from how many different medical schools. What sort of participant data was collected alongside the facilitated discussion?
4. In the Conclusions presented, it seems like a new argument is being advanced which had not previously been introduced anywhere else in the Abstract, nor in the Introduction. If the argument that the education of student doctors is moving away from 'real-world' settings, then some evidence needs to be provided to assert this claim. What is the evidence that UK doctors are not learning on wards anymore? This whole section needs some further thought. Another area had also not previously been mentioned in the Discussion; For example, the second point about training and formative testing, and the overall role of assessment in training was not a feature and it is hard to see the links between any of the data presented and this statement in the Conclusion of the Abstract. The role of assessment in preparing doctors for practice is a completely different issue which doesn't quite emerge from the data presented here and may need some further thinking about how to position this if the authors choose to keep this statement in.
5. The Conclusion presented here reads mor like recommendations rather than a final statement of what was found and explanation of why. The Conclusion that emerged at the end of the paper reflected more holistically on the relationship between education and training which did reflect the results and the arguments made in the Introduction. Perhaps

the Conclusion presented in the Abstract could be more reflective of this also?

Strengths and limitations

- In the second dot point, the authors may have left out a word on line 10-11: "we chose to examine a single that new doctors do" ... should it be "we chose to examine a single *activity* that new doctors do"?
- Also in the second dot point, line 12, consider adding "can" to the sentence "very anxious, and *can* cause considerable harm" to be less equivocal.

Introduction

Although the argument is made well overall, there was insufficient information about the ideographic method to understand and foreground the analysis and results. Ideographic methodology was only introduced briefly in lines 54-60 on page 6 as an example of a wicked problem. Ideographic methodology may be quite new to some readers, so using it to explain something else that may also be novel to readers may not be ideal.

Methods

1. In the section of patient and public involvement, page 7, lines 45-52, the authors should refer the reader to Supplement 1 and the AW program which are really key to understanding the rest of the paper. On my first reading I read the Supplement last and then re-read the paper again much more enlightened. So, an earlier reference to the supplement would enhance the readability of the study for the intended audience.
2. I remain confused however about the whole collection of data and use of facilitators. In the section on Patient and Public Involvement, page 7, the authors state that facilitation provided an opportunity for patients to share their expertise with doctors. However, the CBD described in the Study Design on page 11 indicates the CBD was an opportunity for doctors to unpack their thinking and experiences of the activity (prescribing insulin) with facilitators (who were the trained diabetic patients meant to talk about their own experiences) rather than learn from the facilitator. How did this actually work and were the facilitators trained to have an additional role for this study, one which didn't involve them talking but mor listening to the PGY 1 and 2's?
3. I wanted to know more about the patient facilitators in order to judge the validity of the results against the method. For example, had any of the patient facilitators experienced adverse outcomes themselves? Did PGY1 and 2 doctors know they were going to participate in a CBD with a patient

as a facilitator when they volunteered? What was the likelihood that the facilitators might have been the patient of one of the 66 narratives?

4. I was also interested in the ethics of PGY1 and 2 doctors sharing these stories that involved issues of patient safety with patient facilitators? What were the safeguards for both parties to ensure psychological safety? Was there any restrictions given to the doctors for the timelines in which they should report on an activity? For example, was the time between the experience and then reflecting and recounting it as part of the CBD less than a month? What was the variation there? Was recall bias thought about at all?
5. In the Study design, page 11, it is not clear why the authors relied only on the notes taken by the facilitator to generate the data rather than an audio recording or transcription of the conversation. With 20 facilitators there must have been great variability in the quality of facilitation? How was consistency assured? Were the facilitators also trained in how to debrief the participant? Had any of the doctors been debriefed after an incident or was the retelling of these experiences the first time they had discussed it? Active listening requires constant attention, and so if the facilitator was also trying to write notes that were intended for analysis how might this have impacted the CBD? These are limitations which need to be addressed to provide greater validity of the results. On the surface, it seems like it is a secondary data analysis of secondary data. Why were the trainees' own notes and reflections not used?
6. Page 11, Study Design, lines 32-33, the detailed facilitator notes were sent to the trainee. Was this for fact checking, verification? If so, on how many instances did trainees write back with further clarification and reflections and were these additions incorporated into the notes for analysis?
7. Analytic procedures (page 11): Were there any disagreements between the researchers in interpreting the notes and dimensions? How were these resolved?
8. What was the timeline for data collection? Did the CBD occur over a series of months? Was the expression of interest called just once and did the first 100 reply within a number of weeks or months?

Figure 2

1. Why were 100 chosen? What was the total available sample?
2. If there were 100 chosen for analysis, where does n=66 come from in the Abstract?

3. Were other characteristics of the sample collected? For example, age, gender, previous healthcare experience prior to medicine, school graduated from?
4. To build an understanding of the rigor of this study it would be useful to know which researchers were paired for the first round of data analysis. For example, were CL and FFW in separate pairs? Was there a strategy to the pairing of researchers?
5. What comprises the “raw data” (line 41) Is it the facilitator notes? Were they hand written?
6. It would be useful to include the proforma (line 46) as supplementary data, especially to replicate the methods
7. If the raw data are narratives, the result of a 30 minute CBD, how does this fit into a spreadsheet? More information is needed to visualize and understand this process and it would be useful to see an example of the data extraction spreadsheet

Results

1. Presentation was overall clear and interesting to read
2. After having read the narratives I was wondering how and if the facilitators provided support and feedback. How did the PGY 1 and 2’s ‘cope’ and learn from the experience through the CBD?
3. Were there any differences between PGY1 and 2? Given years of experience on the job?
4. Were the participants’ actions similar across all sites or did some sites provoke more particular types of reactions and actions than others?
5. The results seem to raise a much bigger question about how to change hospital culture in relation to prescribing practice, not necessarily how should curricula change to be better aligned

Discussion

1. In the first paragraph it is stated that curricula need to be more constructively aligned with the practice of medical work. The pace and governance of educational change and innovation may restrict the feasibility of this. Could it be that teaching about complexity in medical work needs to be made more explicit and students need to be taught how to recognize and be attuned to pathophysiological, contextual and social factors. It is hard to demand more workplace learning in contexts which are already resource limited and where supervision is thing on the ground for registrars without adding more medical students into the mix. As the authors’ note, it is a wicked problem, but stating that curricula need to be better aligned seems a little simplistic for a study which has used complexity theory to frame the analysis

2. The authors refer to Sawyer's social emergence theory and Applied mathematics on lines 34-38, page 25. However, a refresh for the reader might be needed here and it may be worth expanding these points to articulate clearly *how* Sawyer and applied mathematics was used
3. The last point on page 25, where it is proposed that healthcare education should be reoriented towards complexity is much more in keeping with the results and arguments presented in the Introduction.
4. The Unanswered Questions section, page 26, provides a great way to frame the study and would be nice to include in the Introduction as it provided a nice argument for the research as well

---

VERSION 1 - AUTHOR RESPONSE

**Reviewers' comments**<br>Reviewer 1 (brown), Reviewer 2 (green)	**Authors' responses; including amendments**
**Overall**	
This is an interesting paper exploring how new doctors manage complexity which is pertinent to medical training and preparation for practice.<br><br>This was a great read, a little challenging in places, and my review provides feedback suggestions it is hoped will improve readability and understanding.<br><br>Thank you for the opportunity to review such a novel and theoretically progressive piece of research. I did read this several times and enjoyed the premise of the paper overall, but I also think there are some areas which need more clarity. The paper will provide a new contribution to the literature and is thought-provoking in how we can best teach in ways that are both psychologically safe but still prepare doctors for the complexity of practice in a variety of contexts. The scope was clearly defined, and this is a much- needed area for investigation as workforce demands change, and universities struggle to adapt their teaching to adequately prepare graduates for practicing in contexts with the increased demands and expectations placed on stressed, burnt-out supervisors who are in short supply.	We appreciate the positive comments, drawing attention to the relevance of the work. We have responded to R2's request for greater clarity in many ways, itemised below. We have also been through the text carefully to shorten sentences and use simpler language, so that the presentation as well as content are clearer.
**Title**	

I think the title should include prescribing/insulin prescribing rather than simply moments of action. With the methodology used the authors comment "we cannot claim that the findings are generalisable" and this should be reflected in the title.	We have included the phrase 'insulin prescribing' in the title. This comment raises an important point that runs through the revision: qualitative research cannot claim to be generalisable. It can, though, claim to be transferable and wouldn't be much use if it weren't. A theme that runs through the revision is the transferability of the findings, extending beyond the specific instance of insulin to prescribing more generally, and (self-evidently) to other clinical actions.
**Abstract**	
Similarly with the objectives in the abstract, the study does not "Provide an in -depth analysis of work that newly qualified doctors do" and this should be more specific.<br><br>The first objective was sound and I think the paper achieved it<br><br>The second objective, and all subsequent discussions of curricula alignment in the paper, seem to be quite a stretch to make and I don't think the authors quite achieved the objective "Propose how undergraduate curricula could be more constructively aligned with the demands of medical work". Upon my first reading, this set up an expectation for some clear guidance about curricula, which was not delivered upon. On my second reading, I could see why this objective was stated and the points made in the Discussion about how simulation belies the complexities of practice in 'real-world' setting and may do a disservice in preparing doctors for practice.<br>However, I think the real gem from the discussion was the point made about the education and training continuum and this could perhaps be the basis for reframing the arguments and rather than 'curriculum alignment' per se.	We agree with the criticism: what we analysed was not the work done, so much as the task as it presented to them, which (as we argue for intravenous fluid therapy in 'methods') exemplifies a task where correct action cannot be accurately predicted (in some cases, at least) at the outset. This highlights the concept of transferability**,** since insulin served as an exemplar, rather than the sole object of the work.<br><br>We agree with the criticism of our use of the term 'constructive alignment'. We no longer use the term and have very carefully articulated in the abstract and later the implications that the education and training continuum should include sufficient immersion experience.<br><br>The objectives now read:<br>1) Analyse in depth an exemplar safety-critical task required of newly qualified doctors (prescribing insulin); 2) Provide transferable insights into how undergraduate education could better educate medical students to meet the demands of practice when they become postgraduate trainees.
2. Social emergence theory also needs to be clearly articulated. It does involve more work on the reader to try to understand the value of it and that type of work detracts from reading the paper to gain new insights that may improve one's educational practice.	Agreed. We also agree with the criticism that mention of applied mathematics was a distractor. We explain, under 'Methods' below, how we have revised the conceptual orientation section for optimum clarity. This includes removing all mention of applied mathematics.
3. The participant numbers are great for a qualitative study of this nature, and I was wondering if they were all UK graduates and from	We have clarified, in methods, that we did not have information about participants' medical schools. We take R2's question as guided by curiosity,

how many different medical schools. What sort of participant data was collected alongside the facilitated discussion?	rather than a critique of the validity of findings. It is not usual for research articles to give details of participants' medical schools. All participants in foundation training will have been required to meet GMC criteria for licensure, irrespective of their school or country of origin.
4. In the Conclusions presented, it seems like a new argument is being advanced which had not previously been introduced anywhere else in the Abstract, nor in the Introduction. If the argument that the education of student doctors is moving away from 'real-world' settings, then some evidence needs to be provided to assert this claim. What is the evidence that UK doctors are not learning on wards anymore? This whole section needs some further thought. Another area had also not previously been mentioned in the Discussion; For example, the second point about training and formative testing, and the overall role of assessment in training was not a feature and it is hard to see the links between any of the data presented and this statement in the Conclusion of the Abstract. The role of assessment in preparing doctors for practice is a completely different issue which doesn't quite emerge from the data presented here and may need some further thinking about how toposition this if the authors choose to keep this statement in.<br><br>5. The Conclusion presented here reads mor like recommendations rather than a final statement of what was found and explanation of why. The Conclusion that emerged at the end of the paper reflected more holistically on the relationship between education and training which did reflect the results and the arguments made in the Introduction. Perhaps the Conclusion presented in the Abstract could be more reflective of this also?	We have, in response to this well-taken criticism, completely revised the conclusion of 'Abstract' to read as follows:<br>New doctors' work includes acting upon indeterminate, emergent situations whose complexity defies rules that are determinate enough to be taught off the job. If new doctors are to perform capably in moments of action, medical students need ample, supervised, situated experience of what it is like to take responsibility in such moments.<br><br>We hope R2 will see this as a defensible conclusion, given the revised line of argument in the article.
**Strengths and limitations**	
In the second dot point, the authors may have left out a word on line 10-11: "we chose to examine a single that new doctors do" … should it be "we chose to examine a single activity that new doctors do"?	We have revised the strengths and limitations to respond to both of R2's criticisms.

• Also in the second dot point, line 12, consider adding “can” to the sentence “very anxious, and can cause considerable harm” to be less equivocal.	
**Introduction**	
My main critique is that … the messaging in the Discussion and Implications were clearer than in the Introduction and so perhaps the authors could think about some re-framing	We have substantially revised the introduction, particularly in response to the comments of R1, in order to strengthen its line of argument. We thank R2 for the positive comment about the discussion; in addition, R1’s critique of our reductionist approach has led to a very substantial revision, going into more depth about existing evidence
Although the argument is made well overall, there was insufficient information about the ideographic method to understand and foreground the analysis and results. Ideographic methodology was only introduced briefly in lines 54-60 on page 6 as an example of a wicked problem.	We can see that the term idiographic caused difficulty, so we have removed it from the manuscript. Rather, we have used more precise descriptions of the study design and procedures to take the place of the term.
One key area of omission is discussion of the medical assistantship, which is required for all students in the UK by the GMC. The assistantship is designed to achieve just these outcomes, of practical application of knowledge in the messy workplace and preparation for practice. There is a literature surround the assistantship eg Kinston et al 2023 which explores similar themes and should be included.<br><br>There is certainly merit in this paper, however …there needs to be more … consideration of the assistantship	We thank R1 for this comment. It created some tension, because we wrote the article for what we believe to be BMJ Open’s target readership – clinician educators internationally, not just in the UK. Viewed in comparison to the US and the Netherlands, the assistantship offers somewhat limited practical immersion, though Kinston’s work from Keele is exemplary. We hope you will be satisfied with the explanation we have now included in the Introduction of the huge variability, of which the assistantship is but one part, in the student to intern/resident transition. We have given the work of Kinston due attention, as requested. (Paragraph 7 of Introduction) and hope that R1 will see that the critical point she raised has helped develop an internationally relevant argument about education for practice.
There is certainly merit in this paper, however for me there needs to be … less reductionist discussion of the current approaches to undergraduate medical education.	We agree with this critique, to which we have responded by going into the literature on clinical decision making, uncertainty, and preparation for practice in more detail and drawing out of it what we feel is a better framing of the problem we addressed. Perhaps our most direct response to R1’s critique is to elaborate the discussion of clinical decision making, citing empirical literature about prescribing education to support our argument.<br>This, we feel, strengthens the introduction as a ‘launchpad’ for the empirical study it precedes. We hope that R1 will see the 5 paragraphs about current approaches as a fitting response.

Suggesting that undergraduate medical education needs more constructive alignment with the workplace is a very generalised statement with the variation in curricula- some of which have extended assistantships, some of which use EPAs to engage students with tasks when they have achieved competence. There is a case put forward for patient safety being better addressed through work-place activity over training, simulation and formative assessment, however most educators would consider that moving through these stages is not a replacement for clinical activity, but rather preparation to ensure safe practice.	Again, we agree with R1's critique and have, in response, removed the concept of constructive alignment from the manuscript as a whole. Our revision accords with R1's thinking by treating workplace experience as but one component of a medical programme, alongside the other components R1 mentions.
from the examples given it appears that this was done by the facilitators, however this raises questions for me regarding the implications for an idiographic approach relying on intact narratives. If these narratives are recorded by a 3rd party they are already being seen through a different lens and the strength of the narrative is depleted. If the 3rd parties have different roles and backgrounds this further muddies the authenticity.	We have substantially revised the manuscript, giving full information about the nature and source of the data. Together with our removal of the term 'idiographic', and a more guarded description of the methodology, we hope that R1 will see that the manuscript now fully accommodates that comment.
I would prefer the details of the precise methods used to be in the body of the text, as this is needed to fully understand the process. The body of the text could be considerably abbreviated.	We have put details of methods in the body of the article. We find the comment that the body of the text could be considerably abbreviated a bit enigmatic because it doesn't indicate where abbreviation might be in order and the two reviewers' criticism have obliged to lengthen the text considerably. We ask the Editor to appreciate that we have had to strike a trade-off between conciseness and completeness of detail, with an increased emphasis at the reviewers behest.
The relevance of applied mathematics to the theoretical construct is unclear, and the reference given is to a popular science book. For me, this adds nothing and could be excluded. The use of social emergence theory is appropriate as a non-reductionist approach for what are certainly areas of complexity	We have removed all reference to applied mathematics.
**Methods**	
There is certainly merit in this paper, however for me there needs to be more clarity of methods,	We agree and list, below, the changes we have made to achieve this.
It is not clear who interviewed the participants- people with lived experience were trained but I'm not clear if it was they who	The BMJ Open required structure for Methods, which prescribed a section on PPI fairly early in methods led to considerable confusion. Because we forefronted the contribution of Patient Advocates, this took attention away

conducted the case based discussions. I would also like to see more clarity on how the discussions were recorded-<br><br>2. I remain confused however about the whole collection of data and use of facilitators. In the section on Patient and Public Involvement, page 7, the authors state that facilitation provided an opportunity for patients to share theirexpertise with doctors. However, the CBD described in the Study Design on page 11 indicates the CBD was an opportunity for doctors to unpack their thinking and experiences of the activity (prescribing insulin) with facilitators (who were the trained diabetic patients meant to talk about their own experiences) rather than learn from the facilitator. How did this actually work and were the facilitators trained to have an additional role for this study, one which didn't involve them talking but mor listening to the PGY 1 and 2's?<br><br>3. I wanted to know more about the patient facilitators in order to judge the validity of the results against the method. For example, had any of the patient<br>facilitators experienced adverse outcomes themselves? Did PGY1 and 2 doctors know they were going to participate in a CBD with a patient as a facilitator when they volunteered? What was the likelihood that the facilitators might have been the patient of one of the 66 narratives?<br><br>5. In the Study design, page 11, it is not clear why the authors relied only on the<br>notes taken by the facilitator to generate the data rather than an audio recording or transcription of the conversation. With 20 facilitators there must have been great variability in the quality of facilitation? How was consistency assured? Were the facilitators also trained in how to debrief the participant? Had any of the doctors been debriefed after an incident or was the retelling of these experiences the first time they had discussed it? Active listening requires constant attention, and so if the facilitator was	from the diverse team, mainly comprising health professionals. This has resulted in two issues becoming conflated, which we have clarified in the revised article and will clarify, also, here.<br><br>These comments called for substantial revision. We hope that the revision, particularly the section of Methods headed 'Study Design' responds appropriately to the criticisms in the following ways.<br><br><u>Role of patient advocates</u>: to avoid an inappropriate emphasis on their role, we have relocated the section referring to patient and public involvement so that it follows 'Study Design'. We have fully explained the training and role of facilitators, making it clear that Patient Advocates were given an opportunity to express their advocacy by being involved, as peers alongside, clinicians, in an interprofessional educational exercise. No patient advocate had, to our knowledge, been involved in an adverse event that was relevant to this project and certainly none of them were patients in the cases discussed. Yes, PGY1s were well aware that a patient advocate might be their facilitator, as we have tried to make even clearer.<br><br><u>Unpacking thinking and experience</u>. This is the means by which facilitated discussions yielded such rich information. The study design section now gives more detail about this.<br><br><u>Why discussions were not recorded</u>: These were authentic, in-depth educational discussions in which, with their consent, new doctors dug deep into their experiences. Facilitators had been trained to empower PGY1/2s to tell their stories and to write notes giving important detail of salient points.<br>We have included the proforma to show the structure that allowed them to do this.<br><br>One thing we learned from this research was that PGY1/2s rarely had opportunities to, in unhurried, psychologically safe settings, 'reflect on action', using Donald Schön's terminology. We agree that active listening requires constant attention and trained facilitators to provide this. As facilitators ourselves, we tended to make brief notes as an aide memoire

also trying to write notes that were intended for analysis how might this have impacted the CBD? These are limitations which need to be addressed to provide greater validity of the results. On the surface, it seems like it is a secondary data analysis of secondary data. Why were the trainees' own notes and reflections not used?<br><br>I was also interested in the ethics of PGY1 and 2 doctors sharing these stories that involved issues of patient safety with patient facilitators? What were the safeguards for both parties to ensure psychological safety? Was there any restrictions given to the doctors for the timelines in which they should report on an activity? For example, was the time between the experience and then reflecting and recounting it as part of the CBD less than a month? What was the variation there? Was recall bias thought about at all?	and elaborate the description of the events once the trainee had left. As for trainees' own notes and reflections, we draw R2's attention to Michael Balint's insistence that doctors, when recounting salient events in group work, did so from memory rather than notes, to facilitate reflection. In our experience, trainees' reflective notes tend to be formulaic. The notes that facilitators made were anything but formulaic, as the results section illustrates.<br><br><u>Timelines and reporting</u>: Participants were invited to present a recent event that was personally salient to them. It is well established that qualitative research may explore, in-depth, events that were recent or in the past. The important thing is the skill of the facilitator in helping a research participant speak. We find it implausible that, in a first sample of 247 stories, and in the in-depth sample of 68 of them, 'recall bias' distorted the findings. As R2 herself notes, this was (by qualitative standards) a huge study and we find it hard to see how our methodology was any less valid than conventional individual and group discussions. The case based discussions were conducted in a climate of unconditional positive regard, which created ideal conditions for authentic data.
The study design and methodology are appropriate for the research question.	We appreciate the affirmation of our design and methodology,.
My main critique is that the methods still lack clarity	We have substantially revised Methods to give greater clarity
1. In the section of patient and public involvement, page 7, lines 45- 52, the authors should refer the reader to Supplement 1 and the AW program which are really key to understanding the rest of the paper. On my first reading I read the Supplement last and then re- read the paper again much more enlightened. So, an earlier reference to the supplement would enhance the readability of the study for the intended audience.	We understand the confusions and appreciate the suggestion that we reference Supplement 1 and the AW programme. We have done our level best to write a clear description that is as helpful to readers as possible.

6. Page 11, Study Design, lines 32-33, the detailed facilitator notes were sent to the trainee. Was this for fact checking, verification? If so, on how many instances did trainees write back with further clarification and reflections and were these additions incorporated into the notes for analysis?	We have clarified that this was for fact checking, and stated that PGY1/2s rarely if ever wrote back with clarification. In qualitative research, it is commonly found that ‘return of findings’ exercises infrequently elicit responses, so we are not surprised by that. The methods section is becoming very long so we hope R2 will excuse us for not commenting on that.
7. Analytic procedures (page 11): Were there any disagreements between the researchers in interpreting the notes and dimensions? How were these resolved?	Reflexive qualitative methodology does not expect consensus to exist a priori, but relies on two reflexive interpreters working together to arrive at a rich interpretation. Please see the section, in Methods, on analytical procedures, which describes our painstaking efforts to arrive at a valid interpretation of the findings
8. What was the timeline for data collection? Did the CBD occur over a series of months? Was the expression of interest called just once and did the first 100 reply within a number of weeks or months?	Act Wisely was funded, initially, by quite a sizeable research grant and, subsequently, by our repeated applications for smaller grants. The programme ran from 2017. After the first author’s retirement in 2020, small numbers of discussions continued, limited by requests from PGY1/2s and our availability to conduct them. As stated, the sample analysed here was an opportunity sample of the first 100 discussions, though we ‘triangulated’ that against the whole dataset of 247 transcribed discussions.
Figure 2<br>1. Why were 100 chosen? What was the total available sample?<br>2. If there were 100 chosen for analysis, where does n=66 come from in the Abstract?<br>3. Were other characteristics of the sample collected? For example, age, gender, previous healthcare experience prior to medicine, school graduated from?	We apologise to R2 that the manuscript was confusing. We have clarified that, in a first phase of analysis, authors reviewed the contents of 247 CBDs, but that, for detailed analysis, we narrowed down to an opportunity sample of 100 CBDs, and that the final analysis included data on the 68 that contained high quality data.

4. To build an understanding of the rigor of this study it would be useful to know which researchers were paired for the first round of data analysis. For example, were CL and FFW in separate pairs? Was there a strategy to the pairing of researchers?<br>5. What comprises the "raw data" (line 41) Is it the facilitator notes? Were they hand written?<br>6. It would be useful to include the proforma (line 46) as supplementary data, especially to replicate the methods<br>. If the raw data are narratives, the result of a 30 minute CBD, how does this fit into a spreadsheet? More information is needed to visualize and understand this process and it would be useful to see an example of the data extraction spreadsheet	We have exhaustively reviewed and revised the 'Study Design' with the express intent of responding to R2's well understood questions. If the description is still deemed inadequate, and there is no limit to the word count of the methods section, we will be pleased to provide additional information, but we doubt that readers would want this.
**Results**	
The results are clearly expressed and the diagrammatic representation is useful. The examples given demonstrate the different facets impacting on practice and decision making.<br>1. Presentation was overall clear and interesting to read	We thank both reviewers for their positive comments.
2. After having read the narratives I was wondering how and if the facilitators provided support and feedback. How did the PGY 1 and 2's 'cope' and learn from the experience through the CBD?	We have mentioned the Distress protocols, and mechanisms in place to support PGY1/2s and facilitators in this article. As stated in this article, two other publications provide answers to those less directly relevant questions.
3. Were there any differences between PGY1 and 2? Given years of experience on the job?	Qualitative research is a blunt tool for making comparisons, and we have now clarified that most participants were FY1s so we cannot answer this question, nor was it ever intended that we would.
4. Were the participants' actions similar across all sites or did some sites provoke more particular types of reactions and actions than others?	Whilst this is an interesting question, it goes beyond the scope of this project to answer it. A far larger sample, and perhaps quantitative methodology would be needed to answer it.
5. The results seem to raise a much bigger question about how to change in hospital culture in relation to prescribing practice, not necessarily how should curricula change to be better aligned	Good point. As noted above, we no longer refer to curricular alignment. We fully agree with the point about culture but, again, comment on this would expand the scope of a tightly argued article.
**Discussion**	

1. In the first paragraph it is stated that curricula need to be more constructively aligned with the practice of medical work. The pace and governance of educational change and innovation may restrict the feasibility of this. Could it be that teaching about complexity in medical work needs to be made more explicit and students need to be taught how to recognize and be attuned to pathophysiological, contextual and social factors. It is hard to demand more workplace learning in contexts which are already resource limited and where supervision is thing on the ground for registrars without adding more medical students into the mix. As the authors' note, it is a wicked problem, but stating that curricula need to be better aligned seems a little simplistic for a study which has used complexity theory to frame the analysis	We take all of those points and have, as noted, removed reference to constructive alignment. We appreciate R2's point about the difficulty of offering more workplace experience within existing curricula, but our findings demands that we put this point forward for discussion, even if it is controversial. In order to accommodate this point, however, we have revised of discussion to make this proposal more guarded.
2. The authors refer to Sawyer's social emergence theory and Applied mathematics on lines 34-38, page 25. However, a refresh for the reader might be needed here and it may be worth expanding these points to articulate clearly how Sawyer and applied mathematics was used<br>3. The last point on page 25, where it is proposed that healthcare education should be reoriented towards complexity is much more in keeping with the results and arguments presented in the Introduction.	We hope that the general clarification of the conceptual orientation of this article will make it unnecessary to revisit Social Emergence theory and we are loath to lengthen the article any more. We hope that, by making the suggested revisions, we have now made the comments on which R2 comments pertinent at this point in the manuscript,
4. The Unanswered Questions section, page 26, provides a great way to frame the study and would be nice to include in the Introduction as it provided a nice argument for the research as well	We appreciate that comment and have done what R2 suggests. Paragraph 2 of our revised Introduction is a direct response to this.
**Other**	
References are largely appropriate, but a high proportion (25%) cite the authors own work.	We have included a lot of references, a proportion of which are, as R1 states, to our own work. Now, 80% of the articles do not cite our work. The main reason we cited this number of articles is that this article is the product of a collaboration between two, independently productive, research groups, working in closely related, fields, whose work is highly cited. This gave us

		the critical mass to do what we hope you regard as very original work. To explain this in more detail:<br>• In the case of the first author, this article is the culmination of a research programme dating back to the millenium, which has progressively carved out the field within which this work was conducted. His group has progressively articulated problems, offered explanations of them, and developed pedagogy to address them: in this case, the Act Wisely pedagogy, of which this is the third report, and the main research output. Many of the articles cited here have been cited frequently by others, so it would be illogical to exclude them solely on the grounds that we wrote them.<br>• Because of the programmatic nature of the research, each article has built on previous ones, gradually building up towards this one. Methodological publications are cited here because, without those citations, we would either have left readers in the dark or further lengthened the manuscript<br>R1 simply commented on the number of articles that reported our own work, but but did not explain why it was detrimental, perhaps because she recognised the reason for it. If, in the editor's view, the proportion of citations of our own work (now reduced) is still too high, please tell us and we will remove the least salient of those we have cited.

VERSION 2 - REVIEW

Reviewer 1

Name Owen, Katherine

Affiliation Warwick Medical School

Date 31-Jul-2025

COI

Thank you to the authors for your attention to the comments made and the clear response. I am satisfied that the changes made have clarified all of the points raised.
